# Direction of Arrival Estimation Based on Received Signal Strength Using Two-Row Electronically Steerable Parasitic Array Radiator Antenna

**DOI:** 10.3390/s22052034

**Published:** 2022-03-05

**Authors:** Mateusz Rzymowski, Krzysztof Nyka, Lukasz Kulas

**Affiliations:** Department of Microwave and Antenna Engineering, Faculty of Electronics, Telecommunications and Informatics, Gdansk University of Technology, Narutowicza 11/12, 80-233 Gdansk, Poland; krzysztof.nyka@pg.edu.pl (K.N.); lukasz.kulas@pg.edu.pl (L.K.)

**Keywords:** Internet of Things (IoT), wireless sensor network (WSN), switched-beam antenna, electronically steerable parasitic array radiator (ESPAR) antenna, received signal strength (RSS), direction-of-arrival (DoA) estimation

## Abstract

In this paper, we present a novel approach to direction-of-arrival (DoA) estimation using two-row electronically steerable parasitic array radiator (ESPAR) antenna which has 12 passive elements and allows for elevation and azimuth beam switching using a simple microcontroller, relying solely on received signal strength (RSS) values measured at the antenna output port. To this end, we thoroughly investigate all 18 available 3D antenna radiation patterns of the antenna measured in an anechoic chamber with respect to radiation coverage in the horizontal and vertical direction and propose a generalization of the power-pattern cross-correlation (PPCC) algorithm involving a high number of multiple calibration planes (MCP) as well as specific combinations of radiation pattern sets. Additionally, a new way of RSS-based DoA estimation accuracy assessment, which involves thorough testing conducted along the elevation direction when RF signals impinging on the antenna arrive from arbitrary θ angles, has been reported in this paper to verify the overall algorithm’s performance. The results obtained for different signal-to-noise ratio (SNR) levels indicate that two-row ESPAR antenna can produce, even for low SNR values, accurate DoA estimation in the horizontal plane without prior knowledge about the elevation direction of the unknown RF signals by using appropriate combinations of only 12 3D antenna radiation patterns.

## 1. Introduction

Wireless sensor networks (WSNs), especially in the Internet of Things (IoT) applications, depend on low-cost wireless transceivers, which are usually integrated with simple microcontrollers to provide the functionality required by different IoT applications [1,2,3]. To increase WSNs capabilities, especially when they are installed in challenging environments, in which connectivity problems may be present due to multipath propagation or presence of interfering radio frequency (RF) signals [3,4], WSN nodes can be integrated with energy-efficient switched-beam antennas (SBAs) [5,6,7,8,9] providing a number of directional radiation patterns. Such patterns can easily be switched electronically by a WSN node’s integrated microcontroller [9,10] in order to improve the overall network performance, e.g., by focusing antenna beams of WSN nodes towards specific directions, and increasing its energy efficiency [9,11,12,13,14,15]. Moreover, for a simple low-cost WSN node, it can enable a capability of direction-of-arrival (DoA) estimation for received RF signals incoming from different WSN nodes belonging to the same network [10].

Electronically steerable parasitic array radiator (ESPAR) antenna is one of the promising SBA designs that can successfully be integrated with a WSN node [10] or a WSN gateway [16]. It relies on a simple, yet very effective, a concept introduced originally by Harrington [17], in which a centrally placed active element is surrounded by 6 passive elements that are connected to variable reactances. In this concept, a directional radiation pattern can be formed by setting the correct values of the reactances, which is well-suited for systems having a single RF transceiver port that could benefit from beamforming capabilities. The first ESPAR antenna designs and prototypes, based on the original Harrington proposal, relied on monopoles connected to varactor diodes [18]. A standard RF transceiver can be connected to the proposed ESPAR antenna output port and, additionally, DoA estimation of impinging RF signals with 2° precision can be performed in the horizontal plane relying solely on received-signal strength (RSS) measurements. However, the necessary bias voltages needed to provide correct values of the reactances at the terminals of 6 passive elements in [18] were produced by 12-bit digital-to-analog converters (DAC) of a digital signal processing (DSP) device. In consequence, this antenna system could not be practically used in energy-efficient wireless sensor systems.

To further adapt the ESPAR antenna proposed in [18] to the applications in WSNs, especially integrated within low-cost battery-powered WSN nodes, a simplified beam steering concept has been proposed [19]. In that approach, 12 passive elements surrounding the active one were used and the varactor diodes were replaced by simple low-cost and low-current integrated single-pole double-throw (SPDT) FET switches to provide load impedances at the terminals of passive elements close to an open or short circuit. Such ESPAR antenna can produce 12 directional radiation patterns which can be formed and rotated in the horizontal plane with 30° discreet steps using digital I/O ports of a simple microcontroller integrated within an RF transceiver. Thus, it can also be used to estimate DoA with 2° precision in an anechoic chamber using power-pattern cross-correlation (PPCC) algorithm, originally proposed in [18], relying solely on RSS measurements at the antenna output port [20]. Therefore, by integrating energy-efficient ESPAR antennas within WSN nodes employing simple and inexpensive RF transceivers that can measure RSS of incoming packets, it was possible to develop WSN nodes capable of performing DoA estimation [10]. Together with accompanying beam focusing functionality it can improve coverage, connectivity and energy efficiency as well as reduce latencies in the whole network [13,14,15,21,22].

One of the main benefits of ESPAR antennas is that, according to existing literature, they can easily be integrated with a WSN node and provide DoA estimation functionality to the node relying solely on RSS measurements gathered for 12 ESPAR antenna radiation patterns [10]. The PPCC algorithm used in [10] for DoA estimation has been originally introduced in [18]. It relies on strong horizontal diversity of ESPAR antenna radiation patterns which are measured beforehand in the horizontal direction in an anechoic chamber and correlates these measurements with RSS values gathered by the WSN node. The original PPCC algorithm has been extended to involve multiple calibration planes (MCP) [23,24] being the radiation patterns measured in an anechoic chamber for different θ directions. It allows 1D DoA estimation of RF signals impinging on the antenna without prior knowledge about their vertical direction, which may vary as the elevation of WSN nodes, may be different in practical implementations [25,26]. Unfortunately, due to the conical shape of ESPAR antenna directional radiation patterns [27], DoA estimation results do not keep the low accuracy levels available for θ = 90° when impinging RF signals have low θ angles [23,24,27]. This effect is especially well pronounced when a reduced number of directional beams are considered. Experiments conducted for a low-profile ESPAR antenna having 8 passive elements in [28] indicate that DoA estimation not only loses accuracy for lower θ angles when 6 or 8 directional radiation patterns are used but also creates ambiguous results making the PPCC-MCP algorithm results unreliable for lower signal-to-noise ratio (SNR) values [28].

To address WSN connectivity challenges in practical industrial installations [25,26] and also possible drone applications [29,30], in which the elevation of WSN nodes may significantly vary, a two-row ESPAR antenna concept has been proposed recently that has 12 passive elements arranged in two rows around the active element. It is capable to create 18 directional radiation patterns covering 3 different elevation directions [31]. The new ESPAR antenna has been designed to provide better connectivity in IoT applications relying on WSN nodes, in which the nodes positions are not restricted to the horizontal plane only, and to provide DoA functionality in such setups. It relies on simple low-power SPDT integrated switches, and therefore, it can easily be incorporated in a WSN gateway or a WSN node. As a consequence, simple elevation and azimuth beam switching allows to create 6 directional beams in the horizontal plane for each of 3 vertical direction.

Combined connectivity and DoA estimation capabilities can highly improve IoT systems involving WSN nodes and gateways installed on the ground in smart factories, buildings and cities [1,2,3,5,6,25,26]. Moreover, it can be used in modern IoT applications involving long-range communication to such objects as unmanned aerial vehicles (UAVs), high-altitude pseudo-satellites (HAPS) or satellite platforms [29,30,32,33], in which the elevation between transceiver and receiver may change in a fast manner. In this regard particularly interesting and growing application area, in which beamforming plays significant role, is providing multiple access and increased security in satellite and aerial integrated networks for IoT communication [34,35,36,37]. In such applications, the proposed two-row ESPAR antenna can successfully provide beamforming capabilities as low weight, and high efficiency have to be considered for practical space applications [38].

Connectivity performance and RSS-based DoA estimation approaches, which are crucial to develop new WSN gateways and nodes integrated with two-row ESPAR antenna, have not been investigated in the original publication [31]. According to the authors’ knowledge, they are also not currently available in the literature. Therefore, the main contributions of this paper are:

- In-depth analysis of the antenna from the connectivity perspective with respect to possible beam steering in horizontal and elevation directions;
- Presentation of an approach for DoA estimation relying solely on RSS values gathered at the antenna output, which is a prerequisite for energy-efficient WSN nodes having DoA functionality [9,10], that is suitable for the antenna and can provide acceptable DoA estimation results also for low θ angles and are free from ambiguities for lower SNR values;
- Proposal of a detailed DoA algorithm performance testing method for more accurate DoA estimation accuracy assessment that involves all θ angles to address strong error variation at low θ angles.

The rest of this paper is organized as follows: in Section 2 the two-row ESPAR antenna is described together with its simulated and measured radiation patterns as well as their influence on the overall connectivity in WSN-based IoT setups, in which the positions of nodes are not restricted to the horizontal plane only. Section 3 describes a PPCC-MCP algorithm and its proposed generalization for the usage with the two-row ESPAR antenna. Results obtained using a new RSS-based DoA estimation accuracy assessment involving thorough testing conducted along the elevation direction are presented in Section 4, while Section 5 presents concluding remarks.

## 2. Two-Row ESPAR Antenna

### 2.1. Antenna Design

**Figure 1:**
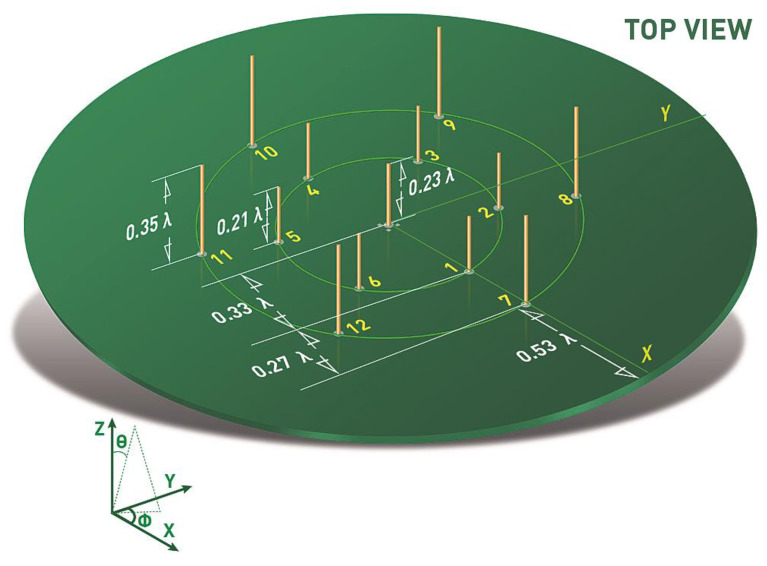
Two-row ESPAR antenna model.

The proposed antenna concept has been discussed in [31] and is presented in Figure 1. It was aimed to achieve a low-cost reconfigurable antenna that is able to provide maximal angular radiation coverage by steering the beam in elevation and azimuth. The active quarter-wave monopole is located in the center of the metallic ground plane realized on the top of a dielectric substrate and surrounded by 12 passive radiators symmetrically arranged in two circular rows. The active radiator is fed coaxially with RF signal, while the passive radiators can be open or shorted to the ground plane by changing the state of the SPDT switches. Forming the radiation pattern is realized by setting proper configuration of the passive elements that become reflectors when shorted to the ground or directors when opened. Such an approach simplifies the beam steering control that can be realized with external microcontroller or transceiver having its general purpose input/output (GPIO) lines connected to the RF switches. As a result, each radiation pattern corresponds to a steering vector $V=\left[v_{1},\;v_{2},\ldots ,v_{s},\ldots ,v_{12}\right]$, in which $v_{s}$ denotes the state of *s*th passive element: $v_{s}=1$ for open and $v_{s}=0$ for the shorted one. The antenna was optimized to provide three sets of directional radiation patterns with different inclination angles $\theta_{max\_up}=46{}^{\circ}$, $\theta_{max\_down}=56{}^{\circ}$, and $\theta_{max\_mid}=52{}^{\circ}$ obtained for three different steering vectors sets. The symmetrical design allows rotating each beam by 360°, which means that it is possible to steer the radiation pattern in both, azimuth and elevation. Therefore, for each elevation direction, 6 directional radiation patterns having maximum in azimuth at 30°, 90°, 150°, 210°, 270°, 330° are available for steering vectors $V_{UP}^{\varphi_{max}}$, $V_{DOWN}^{\varphi_{max}}$, and $V_{MID}^{\varphi_{max}}$, where $\varphi_{max}=\left\{30,\;90,\;150,\;210,\;270,\;330\right\}$ degrees. The antenna was designed and optimized in Altair FEKO simulation tool to operate at center frequency 2.44 GHz and the detailed design procedure including optimization goals was described in [31].

### 2.2. Realized Antenna 

**Figure 2:**
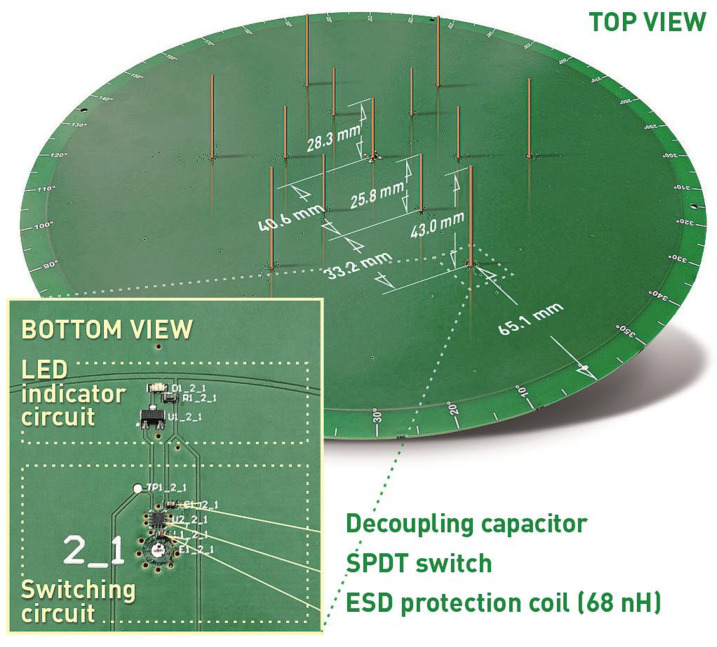
Fabricated two-row ESPAR antenna.

**Figure 3:**
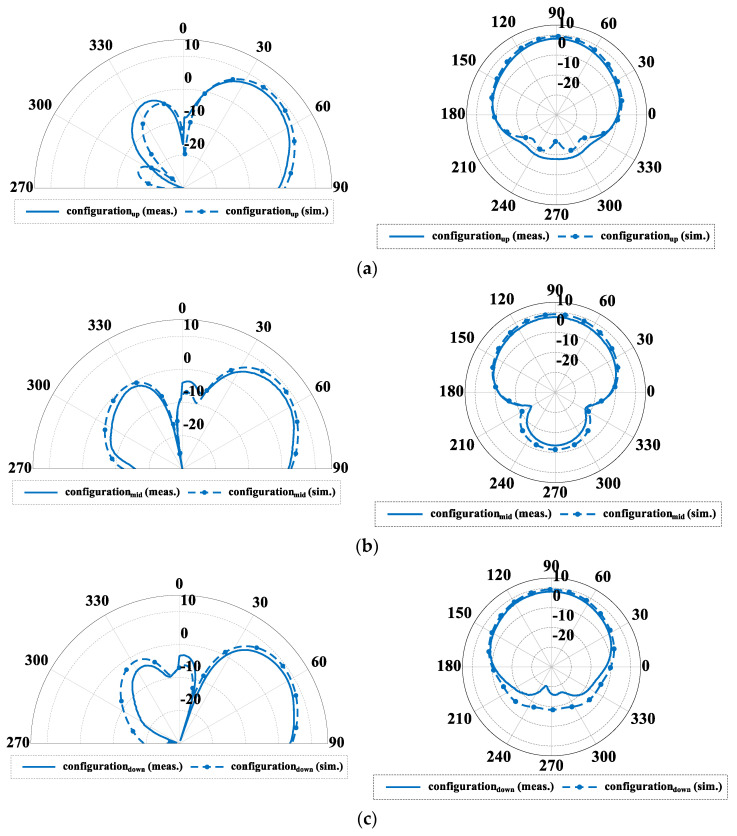
Simulated and measured antenna radiation patterns at 2.44 GHz in the elevation plane (for *φ* = 90°) and in the horizontal plane for three configurations (see text for explanations): (**a**) $V_{UP}^{90}$ (maximum at $\theta_{max\_up}=46{}^{\circ}$ ), (**b**) $V_{MID}^{90}$ (maximum at $\theta_{max\_mid}=52{}^{\circ}$ ), and (**c**)$\;V_{DOWN}^{90}$ (maximum at $\theta_{max\_down}=56{}^{\circ}$).

**Figure 4:**
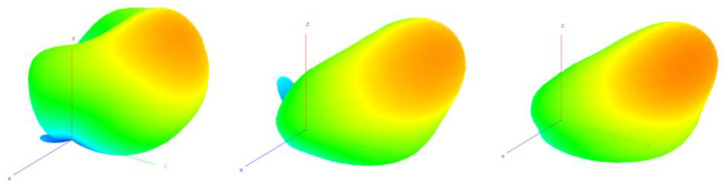
Measured 3D radiation patterns for each considered configuration with different inclination angles: $\theta_{max\_up}$, $\theta_{max\_mid}$, $\theta_{max\_down}$ (from left to right).

**Figure 5:**
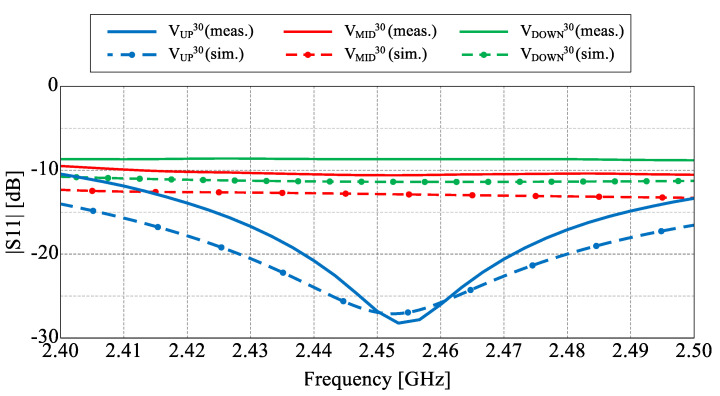
Values of |S11| for all three considered configurations.

The fabricated antenna is presented in Figure 2. 1.55 mm thick FR4 substrate with metalized top layer has been used as a PCB for the switching circuitry and the ground plane of the antenna. The RF connector and switching circuits, as well as LED indicators have been located on the bottom layer as illustrated in Figure 2. The switching circuits employ NJG1681MD7 SPDT switches providing a trade-off between high isolation, low insertion losses and power consumption in comparison to PIN diodes or varactors [28,39,40]. To provide proper performance and avoid eventual RF signal degradation, a decoupling capacitor and ESD protection coil have been placed in close proximity of the switch pins. An LED indicator has been located near each switching circuit to indicate the actual state of the switch (lights when the circuit is in open state). The steering is controlled by an external microcontroller connected to the board. The fabricated antenna has been measured and verified with the simulation model. The assumed three groups of similar beam configurations with different angle tilt in elevation have been simulated, measured and gathered in Figure 3 and Figure 4, while the main antenna parameters for each configuration at the center frequency have been summarized in Table 1. The simulated results have been confirmed by the measurements and only small discrepancies in the backward radiation level can be observed. Measured gain for all characteristics is on a similar level around 8 dB. The input impedance matching results for all configurations are presented in Figure 5 which shows that the achieved |S11| values are close or below −10 dB. The large difference of impedance matching between configurations is a trade-off to achieve the highest possible difference of maximal direction of the radiation pattern in elevation.

### 2.3. Antenna Radiation Performance Analysis 

**Figure 6:**
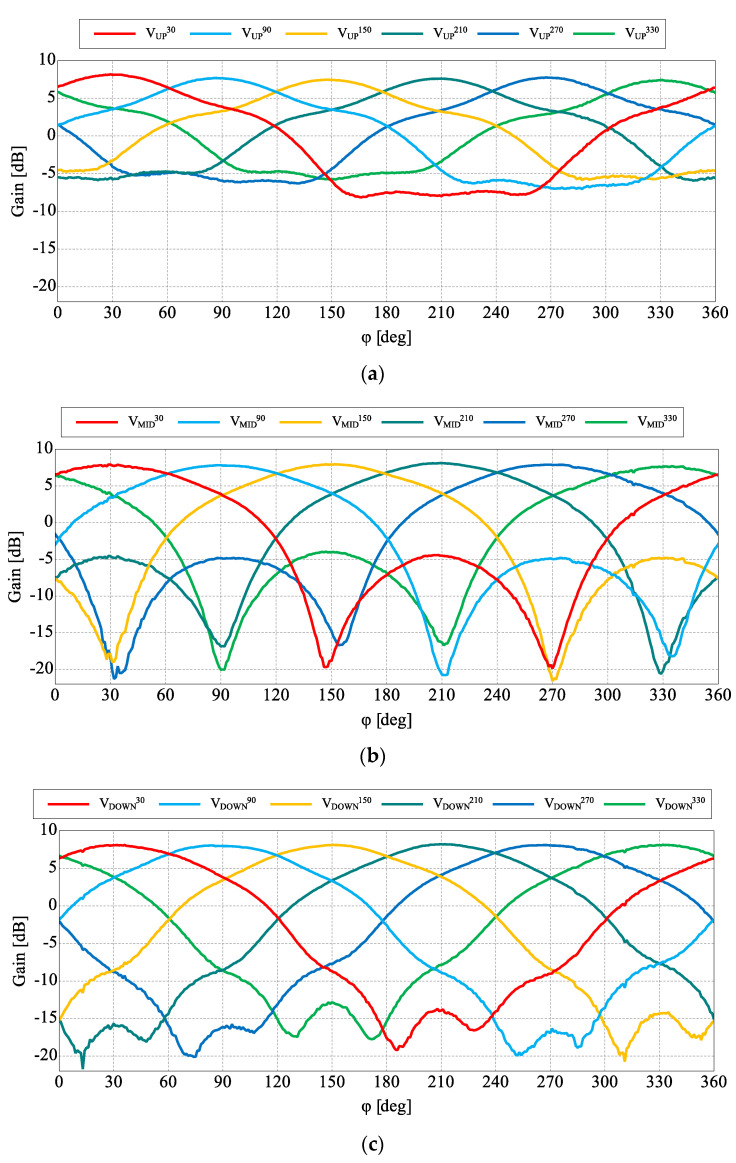
Measured radiation patterns for each configuration in all directions for steering vectors: (**a**) $V_{UP}^{\varphi_{max}}$, (**b**) $V_{MID}^{\varphi_{max}}$, and (**c**)$\;V_{DOWN}^{\varphi_{max}}$.

**Figure 7:**
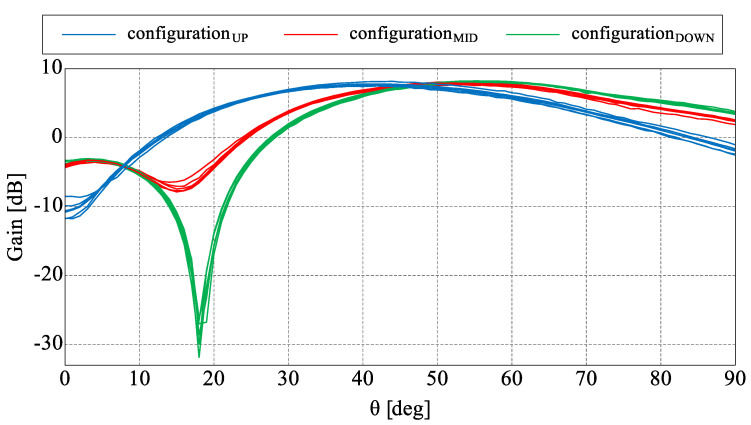
Measured radiation patterns in elevation plane for all directions.

The main goal of the proposed antenna was oriented towards connectivity improvement by increased angular coverage of the directional radiation pattern that can be switched in both, azimuth and elevation. For this reason, a more detailed analysis of the antenna radiation capabilities has to be considered. In Figure 4, measured 3D radiation patterns have been presented which proves the similarity of the directional beams for all three sets. For detailed analysis and comparison, the measured radiation patterns for all configurations in their maximal directions in both, horizontal and elevation plane have been presented in Figure 6 and Figure 7. The similarity of the radiation pattern sets in horizontal plane have been confirmed for all directions of the antenna. The slight differences can be noticed in half power beam width (HPBW) where the values vary from 88° to 100° and in case of the sidelobes level (SLL), 10 dB vs 25–28 dB when comparing UP configuration to MID and DOWN configurations. In Figure 7, one can see the measured radiation patterns in elevation plane. The tilt difference and angular coverage for each set is visible. HPBW in the elevation plane is almost the same for all radiation pattern sets being around 45 degrees. It can be observed that the results are repeatable for all measured configurations.

**Figure 8:**
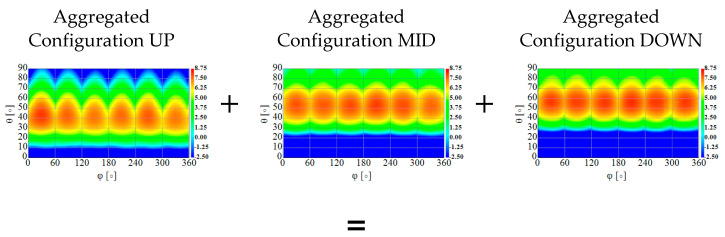

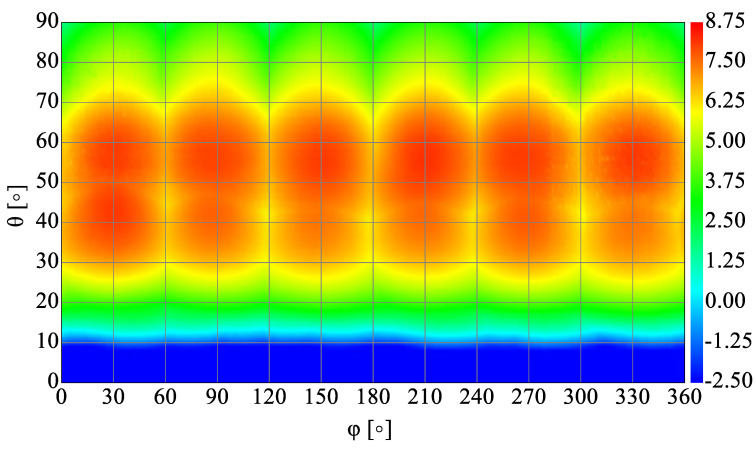
Aggregated gain for all measured configurations.

**Figure 9:**
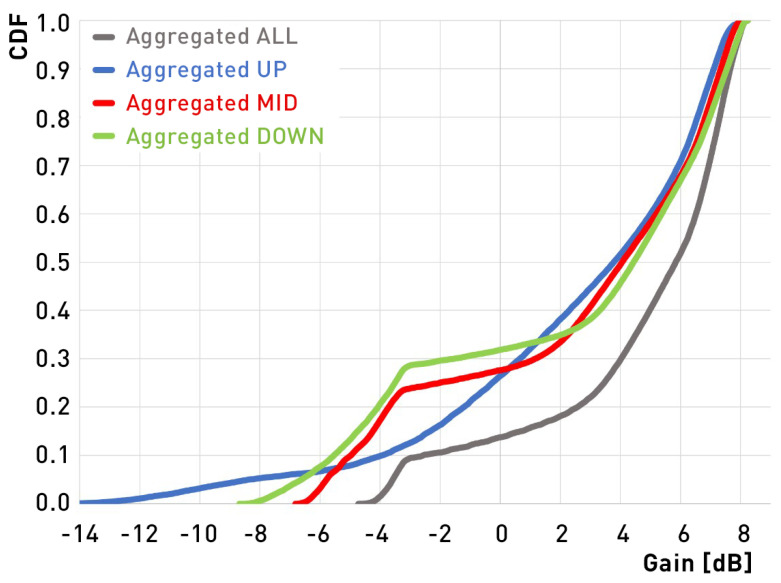
Cumulative Distribution Function of the aggregated gain for different sets of characteristics.

To assess the angular coverage of the antenna that include all possible beam configurations, aggregated radiation patterns need to be considered. This form of presentation is known in the context of time-modulated arrays and 3D coverage analysis for 5G applications where the aggregated characteristic consists of all available antenna radiation patterns to show the complete spatial coverage providable by the antenna [41]. Aggregation is selecting the highest gain values for each angle from the available radiation pattern sets. In the discussed construction the aggregated radiation pattern consists of 18 characteristics: 6 for UP configuration, 6 for MID configuration and 6 for DOWN configuration. In Figure 8, it can be seen that the angular coverage range in elevation for aggregated gain above 0 dB starts from θ≈10°. It means that only limited space under the antenna can be considered as a blind zone. To emphasize the difference in the antenna spherical coverage dependent on the selected beam configurations, a cumulative distribution function (CDF) of aggregated gain can be used [42]. In Figure 9, a CDF for the discussed antenna is presented and aggregated beams for each configuration are compared with aggregated function of all 18 radiation patterns. For example, it can be seen that for 50% of the hemisphere the aggregated gain value is 2 dB higher when considering all 18 radiation patterns instead of only one of the selected configurations. The results clearly indicate higher angular coverage of the antenna when all configurations are used.

## 3. RSS-Based DoA Estimation for Two-Row ESPAR Antenna

Power-pattern cross-correlation algorithm is one of the most practical methods to determine unknown directions of RF signals impinging on an ESPAR antenna [10,18,19,20]. It relies solely on RSS measurements gathered at the antenna output port and uses simple correlation coefficient calculation [18]. When written as matrix-vector operations, they can easily be integrated with a simple microcontroller and provide DoA estimation functionality to the WSN node [10]. In its original implementation, the algorithm relies on ESPAR antenna radiation patterns measured in an anechoic chamber only in the horizontal direction [18]. In consequence, it makes DoA estimation results accurate in the *θ* = 90° direction (i.e., the horizontal plane) with 2–4° precision being the maximum DoA estimation error [18,19,20]. When the directions of RF signals impinging on an ESPAR antenna are different, one may expect an accuracy drop. It has been reported that for low values of θ angle of RF signals impinging on the antenna the maximal error, referred to as precision, of 1D DoA estimation performed in the horizontal direction can easily reach 13° due to the fact that the shape of ESPAR antenna radiation patterns with respect to φ angles gradually changes for *θ* > 90° [23]. To improve the overall accuracy for the *θ* angles different than 90°, one can employ multiple calibration planes (MCP) [23,24] in the original PPCC formulation, which relies on a single *θ* = 90° calibration plane [18].

PPCC-MCP algorithm uses ESPAR antenna radiation patterns measured in chosen vertical directions. In the first implementation, all ESPAR antenna radiation patterns were measured in an anechoic chamber at *θ* = 90° and *θ* = 45° and, based on such two calibration planes, it was possible to maintain 4° precision for RF signals impinging from angles between *θ* = 50° and *θ* = 90° [23]. Involvement of 9 calibration planes, namely {*θ*_1_ = 10°, *θ*_2_ = 20°, …, *θ*_9_ = 90°}, provides 6° 1D DoA precision for RF signals impinging from 9 test vertical angles spanning equally between *θ* = 10° and *θ* = 90°. However, it has been shown that PPCC-MCP algorithm accuracy is sensitive to correct placement of calibration planes positions [24], while obtained results depend on testing setup [28] parameters, especially the choice of testing directions in horizontal and vertical planes as well as SNR values set for testing signals [24,28]. Moreover, the existing PPCC formulation was created to provide DoA results based on a number of directional radiation patterns, while new ESPAR antennas can have a number of possible radiation patterns that can be used in DoA estimation [32,33] having different performance, especially when testing signals may come from multiple horizontal and vertical directions. Therefore, to be used for DoA estimation together with new ESPAR antennas, including the two-row ESPAR antenna proposed in [31], PPCC-MCP algorithm and associated DoA testing methods have to be generalized.

### 3.1. PPCC-MCP Algorithm

PPCC algorithm which allows DoA estimation based on ESPAR antenna radiation patterns measured in an anechoic chamber relies on a cross-correlation coefficient between the measured radiation patterns and RSS values recorded for each directional antenna radiation pattern [18]. The cross-correlation coefficient for the ESPAR antenna having 12 directional beams has the following form:

(1) $$\Gamma \left(\varphi \right)=\frac{\sum_{n=1}^{12}\left(P\left(V_{max}^{n},\varphi \right)\;Y\left(V_{max}^{n}\right)\right)}{\sqrt{\sum_{n=1}^{12}P{\left(V_{max}^{n},\varphi \right)}^{2}}\sqrt{\sum_{n=1}^{12}Y{\left(V_{max}^{n}\right)}^{2}}}$$

where $V_{max}^{n}$ is ESPAR antenna’s steering vector that allows to create n-th directional radiation pattern, $P\left(V_{max}^{n},\varphi \right)$ are directional ESPAR antenna radiation patterns measured during the calibration phase in an anechoic chamber in the azimuth plane for 0°≤φ<360°, $Y\left(V_{max}^{n}\right)$ are RSS values measured at the antenna output port for all 12 directional radiation patterns during the DoA estimation phase for an unknown RF signal, and Γ(*φ*) is the cross-correlation coefficient, which have its highest value associated with the estimated direction of arrival angle $\hat{\varphi }$ [18].

In practical implementations, the angular step precision Δ*φ* = 1° is commonly used during the calibration phase, and therefore, the cross-correlation coefficient can be written in a convenient vector form as [19]:

(2) $$g=\frac{\sum_{n=1}^{12}\left(p^{n}Y\left(V_{max}^{n}\right)\right)}{\sqrt{\sum_{n=1}^{12}(p^{n}\circ p^{n})}\sqrt{\sum_{n=1}^{12}Y{\left(V_{max}^{n}\right)}^{2}}}$$

where vector $p^{n}={\left[p_{1}^{n},\;p_{2}^{n},\cdots ,\;p_{I}^{n}\right]}^{T}$, where the superscript *^T^* is the vector transpose operator, contains I=360 measured discreet values of $P\left(V_{max}^{n},\varphi \right)$ and ‘∘’ denotes the element-wise product of vectors. In result, the cross-correlation coefficient $g={\left[\Gamma \left(\varphi_{1}\right),\;\Gamma \left(\varphi_{2}\right),\;\ldots ,\Gamma \left(\varphi_{I}\right)\right]}^{T}$ is also a vector with I=360 entries being discretized values of the correlation coefficient Γ(φ) for every considered value of φ in $\varphi ={\left[\varphi_{1},\;\varphi_{2},\;\ldots ,\;\varphi_{I}\right]}^{T}={\left[0{}^{\circ},\;1{}^{\circ},\;\ldots ,\;359{}^{\circ}\right]}^{T}$ and $\hat{\varphi }$ corresponds now to the maximum value of g. One should note, however, that as only a single calibration plane at *θ* = 90° is used to measure directional ESPAR antenna radiation patterns in an anechoic chamber during the calibration phase, cross-correlation operation Equation (2) will produce the most accurate results when RF signals impinging on the antenna arrive from *θ* = 90° vertical angle, which is aligned with the calibration plane. The results will significantly deteriorate for lower *θ* angles as the shape of ESPAR antenna radiation patterns gradually changes for *θ* > 90° [23,24].

To further extend PPCC algorithm and improve its accuracy in situations when *θ* angles of RF signals impinging on the antenna are different than 90°, PPCC-MCP algorithm, which allows to incorporate higher number of calibration planes, has been introduced [24]. It is based on the following cross-correlation coefficient:

(3) $$g_{\theta }=\frac{\sum_{n=1}^{12}\left(p_{\theta }^{n}\;Y\left(V_{max}^{n}\right)\right)}{\sqrt{\sum_{n=1}^{12}\left(p_{\theta }^{n}\circ p_{\theta }^{n}\right)\;}\sqrt{\sum_{n=1}^{12}Y{\left(V_{max}^{n}\right)}^{2}\;}}$$

where, for the considered number of M calibration planes Equation (3), vector $p_{\theta }^{n}={\left[\;{\left(p_{\theta_{1}}^{n}\right)}^{T},\;{\left(p_{\theta_{2}}^{n}\right)}^{T},\;\cdots ,\;{\left(p_{\theta_{M}}^{n}\right)}^{T}\;\right]}^{T}$ of length I·M contains ESPAR antenna’s radiation pattern values measured at $\left\{\theta_{1},\;\theta_{2},\;\ldots ,\theta_{M}\right\}$ angles in elevation direction and vector $g_{\theta }$ of length I·M contains PPCC-MCP cross-correlation coefficient in a vector form. As every discretized radiation pattern $p_{\theta_{m}}^{n}$, where m={1, 2, …, M}, corresponds to discretized values of *φ* in the same vector φ, the values in $p_{\theta }^{n}$ correspond to those in the vector $\varphi_{\theta }={\left[\varphi_{\theta_{1}}^{T},\;\varphi_{\theta_{2}}^{T},\cdots ,\;\varphi_{\theta_{M}}^{T}\right]}^{T}={\left[\varphi^{T},\;\varphi^{T},\cdots ,\;\varphi^{T}\right]}^{T}$ [24,28]. In consequence, the estimated DoA angle $\hat{\varphi }$ is now a value in $\varphi_{\theta }$ that corresponds to the highest value in $g_{\theta }$ [28]. It should be underlined, however, that both the number of calibration planes and choice of their *θ* angles highly influence the overall DoA estimation accuracy when RF signals impinging on the antenna arrive from arbitrary *θ* angles [23,24,28,34].

### 3.2. Generalized PPCC-MCP Algorithm for Two-Row ESPAR Antenna

The vector form of the PPCC algorithms in Equations (2) and (3) is appropriate for implementation in a WSN node’s microcontroller to compute DoA estimation based on RSS measured for incoming packets. It is also straightforward to use such a node with ESPAR antennas having different number of directional radiation patterns or other switched beam antennas [28]. However, it is possible to create many different directional radiation patterns in ESPAR antennas and also patterns that do not have a clearly shaped directional beam. For an ESPAR antenna having 12 passive elements designed for vehicle-to-everything (V2X) applications in 802.11p frequency band, due to performance of microwave switches used at the terminals of passive elements in 5.9 GHz frequency band, it was possible to form 5 different directional radiation patterns types [32] having different beam parameters in the horizontal plane, e.g., gain, HPBW and sidelobe level (SLL), as well in the vertical plane. As every radiation pattern type can be rotated in the horizontal plane to form 12 directional beams, PPCC and PPCC-MCP algorithms can successfully be used in RSS-based DoA estimation [33]. Unfortunately, although such estimation could involve different radiation pattern types in a single estimation, to further increase the overall accuracy, appropriate and convenient PPCC-MCP algorithm formulation has not been created so far. Similarly, among 18 available two-row ESPAR antenna radiation patterns, there exist 6 groups having similar main beam direction in the horizontal plane with differences radiation pattern shape in elevation.

To perform RSS-based DoA estimation based on radiation patterns available for two-row ESPAR antenna, one has to create a generalized PPCC-MCP algorithm that can easily be implemented within simple WSN nodes and involves all possible radiation patterns. Due to their spatial characteristics, it is possible to increase the overall accuracy of the estimation while mitigating the necessity of correct placement of calibration planes. As the two-row ESPAR antenna radiation patterns, which are created using associated steering vectors $V^{n}$, exhibit certain spatial performance, including gain, SLL, direction of maximal radiation and HPBW, in both horizontal and vertical directions, we propose rewriting Equation (3) in the following general form:

(4) $$g_{\theta_{ALL}}=\frac{\sum_{n=1}^{N}\left(p_{\theta_{ALL}}^{n}\;Y\left(V^{n}\right)\right)}{\sqrt{\sum_{n=1}^{N}\left(p_{\theta_{ALL}}^{n}\circ p_{\theta_{ALL}}^{n}\right)\;}\sqrt{\sum_{n=1}^{N}Y{\left(V^{n}\right)}^{2}\;}}$$

Total number of radiation patterns used in the DoA estimation and $n=\left\{n_{1},\;n_{2},\;\ldots ,\;n_{N}\right\}$ are numbers of chosen steering vectors $V^{n}$ associated with specific two-row ESPAR antenna radiation patterns $p_{\theta_{ALL}}^{n}$. Since $\theta_{ALL}$ means that all M = 90 possible angles in elevation direction, namely $\left\{\theta_{1}=90{}^{\circ},\;\theta_{2}=89{}^{\circ},\;\ldots ,\theta_{90}=1{}^{\circ}\right\}$, are used as calibration planes, a high-precision turntable in the anechoic chamber is required. In consequence, vectors $p_{\theta_{ALL}}^{n}$, $g_{\theta_{ALL}}$, $\varphi_{\theta_{ALL}}$ will be much longer with the total length I·M=32400, which will make PPCC-MCP calculation more time consuming. However, the overall DoA estimation accuracy of the proposed generalized PPCC-MCP algorithm is no longer dependent on the choice of number and placement of calibration planes. Therefore, the overall DoA estimation accuracy will not be affected when RF signals impinging on the antenna arrive from arbitrary *θ* angles.

## 4. Results and Discussion

### 4.1. Mesurement Setup

**Figure 10:**
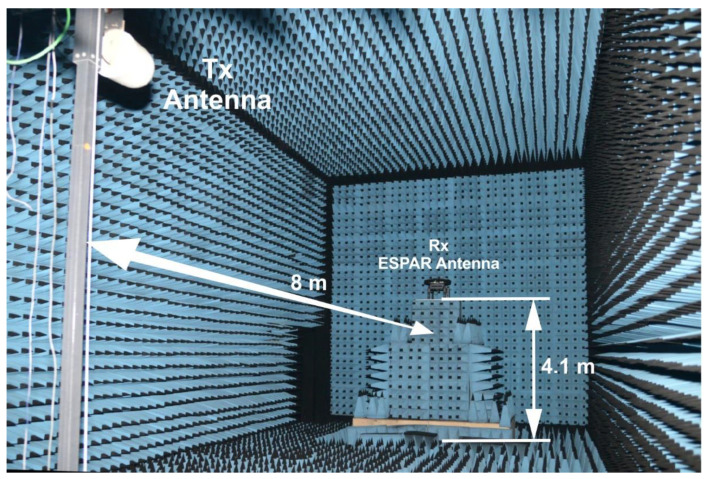
Anechoic chamber measurement setup used for the calibration phase of the proposed generalized PPCC-MCP algorithm for two-row ESPAR antenna.

To verify DoA estimation performance of the two-row ESPAR antenna using the generalized PPCC-MCP method 18 available antenna radiation patterns were measured in our 11.9 m × 5.6 m × 6.0 m anechoic chamber, shown in Figure 10, at 2.44 GHz using P9374A Keysight Streamline USB Vector Network Analyzer. The antenna was mounted at *H* = 4.1 m on a precise and equipped with a digital encoder turntable, which is able to set any 2D position with 0.1° precision in both horizontal and vertical directions. The transmitting antenna was placed on a pole stand at the same height *H* = 4.1 m, 8 m from the two-row ESPAR antenna. Every radiation pattern was measured with the angular step Δφ=1° in the horizontal plane for 0°≤φ<360° and with the angular step Δθ=1° in the vertical plane for 0°<θ≤90°. As a result, *I* = 360 calibration points were produced for every elevation plane and M = 90 calibration planes were created, namely $\left\{\theta_{1}=90{}^{\circ},\;\theta_{2}=89{}^{\circ},\;\ldots ,\theta_{90}=1{}^{\circ}\right\}$.

### 4.2. Deatiled DoA Testing Method

To examine DoA estimation accuracy, measured calibration planes were imported to Matlab, where 10 snapshots of sinusoidal test signal were generated for all considered test directions $\left(\varphi_{t},\theta_{t}\right)$ of impinging RF signals. During each test, as snapshots have to be received at the antenna’s output [19,20,23,33], all sinusoidal test signals were multiplied by two-row ESPAR antenna radiation patterns values measured at appropriate test directions. Then, additive white Gaussian noise (AWGN) was added to obtain a required signal-to-noise ratio (SNR) and RSS value was calculated. Thus, the SNR value has not to be measured as it is determined by setting the appropriate value of spectral power density of numerically added AWGN. This reflects the measurement procedure presented in [20]. In result, for every considered test direction $\left(\varphi_{t},\theta_{t}\right)$ an RSS value has been obtained for all considered two-row ESPAR antenna radiation patterns.

To compare the results with those already available in the literature, the test signal directions have to be set with discrete angular steps equal to $\Delta \varphi_{t}=10{}^{\circ}$ and $\Delta \theta_{t}=10{}^{\circ}$ in horizontal and elevation directions, respectively [24,28,33]. In consequence, one obtains 36 test horizontal directions $\varphi_{t}\in \left\{0{}^{\circ},\;10{}^{\circ},\;\ldots ,350{}^{\circ}\right\}$ for 9 elevation angles $\theta_{t}\in \left\{90{}^{\circ},\;80{}^{\circ},\;\ldots ,10{}^{\circ}\right\}$, which results in 324 testing points. Then, for every elevation angle, PPCC-MCP algorithm is used and, based on obtained 36 estimated direction of arrival angle $\hat{\varphi }$ values, root-mean-square error (RMSE) is calculated together with precision being the maximal DoA estimation error value within the plane. However, it has been observed in [43] that when $\Delta \varphi_{t}=5{}^{\circ}$ and $\Delta \theta_{t}=5{}^{\circ}$ is used, more accurate test results can be provided. Therefore, additional new testing directions, especially in elevation, reveal lack of accuracy of DoA estimation using ESPAR antenna together with PPCC-MCP algorithm for low θ angles when *M* = 9 calibration planes are used. As it was overlooked in [24], to verify DoA estimation performance in a detailed way for the proposed two-row ESPAR antenna and generalized PPCC-MCP algorithm, the number of testing points has to be further increased. To this end, we propose to use detailed DoA testing method that involve $\Delta \varphi_{t}=1{}^{\circ}$ and $\Delta \theta_{t}=1{}^{\circ}$ in horizontal and elevation directions correspondingly. In result, 360 test horizontal directions $\varphi_{t}\in \left\{0{}^{\circ},\;1{}^{\circ},\;\ldots ,359{}^{\circ}\right\}$ for 90 elevation angles $\theta_{t}\in \left\{90{}^{\circ},\;89{}^{\circ},\;\ldots ,1{}^{\circ}\right\}$ are created and, consequently, 90 RMSE and 90 precision values will be available for each plane.

### 4.3. DoA Estimation Results

To verify DoA estimation performance of the two-row ESPAR antenna using the generalized PPCC-MCP algorithm, which uses all 90 available calibration planes, *N* = 18 available antenna radiation patterns were divided into 3 separate groups having different angle tilt in elevation, as shown in Table 1 and Table 2. In result, $n_{up}=\left\{1,\;2,\;3,\;4,\;5,6\right\}$, $n_{down}=\left\{7,8,9,10,11,12\right\}$, and $n_{mid}=\left\{13,14,15,16,17,18\right\}$ were created as sets of steering vectors numbers associated with the specific two-row ESPAR antenna radiation patterns that can be created when particular steering vectors sets, namely $V^{n_{up}}=\left\{V^{1},\;V^{2},\;V^{3},\;V^{4},\;V^{5},V^{6}\right\},$ $V^{n_{down}}=\left\{V^{7},\;V^{8},\;V^{9},\;V^{10},\;V^{11},V^{12}\right\},$ and $V^{n_{mid}}=\left\{V^{13},\;V^{14},\;V^{15},\;V^{16},\;V^{17},V^{18}\right\}$, are used. It allows verification of DoA estimation performance when $g_{\theta_{ALL}}$ is calculated using each of steering vectors set separately but also when they are combined in order to find the most appropriate combination for the two-row ESPAR antenna.

**Figure 11:**
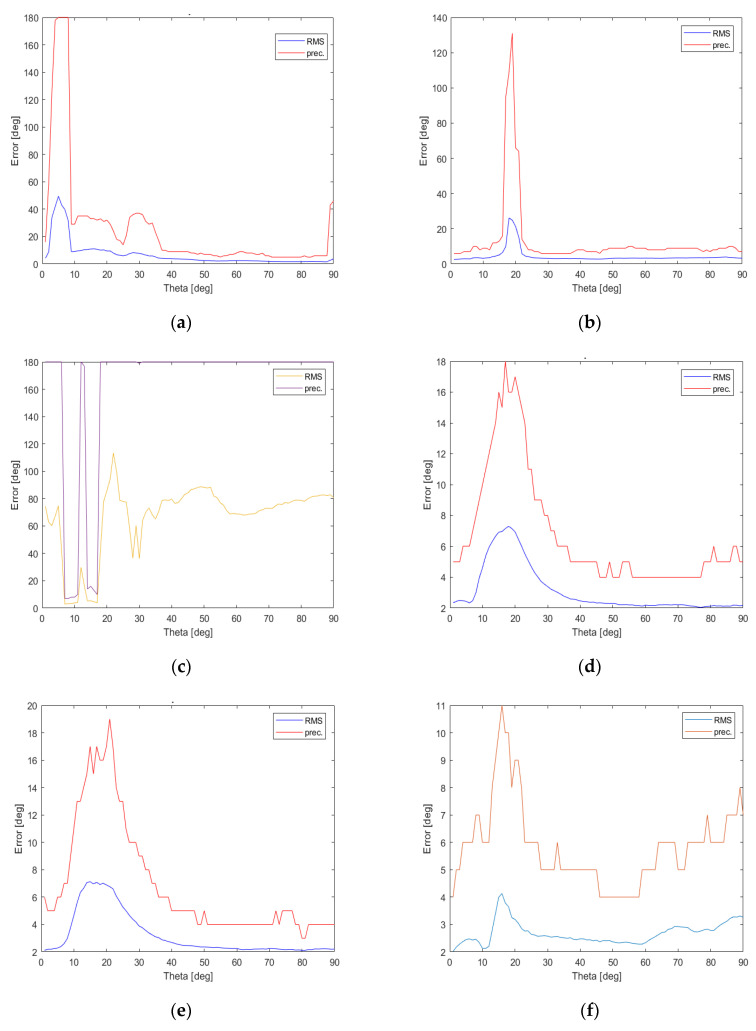
RMSE and precision values calculated using the proposed detailed DoA testing method, which involves all elevation angles during testing process, for the generalized PPCC-MCP algorithm relying on combinations of specific steering vector sets: (**a**) $\left\{V^{n_{up}}\right\}$; (**b**) $\left\{V^{n_{mid}}\right\}$; (**c**) $\left\{V^{n_{down}}\right\}$; (**d**) $\left\{V^{n_{up}},V^{n_{mid}}\right\}$; (**e**) $\left\{V^{n_{up}},V^{n_{down}}\right\}$; (**f**) $\left\{V^{n_{mid}},V^{n_{down}}\right\}$.

**Figure 12:**
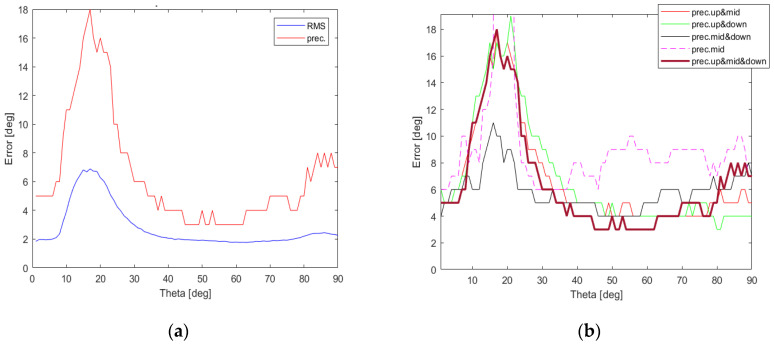
RMSE and precision values calculated using the proposed detailed DoA testing method, which involves all elevation angles during testing process, for the generalized PPCC-MCP algorithm relying on all possible steering vectors $\left\{V^{n_{up}},V^{n_{mid}},V^{n_{down}}\right\}$ (**a**) together with precision value comparison for combinations of sets giving the most accurate results (**b**).

To test DoA estimation accuracy in a detailed way, 360 test horizontal directions $\varphi_{t}\in \left\{0{}^{\circ},\;1{}^{\circ},\;\ldots ,359{}^{\circ}\right\}$ for 90 elevation angles $\theta_{t}\in \left\{90{}^{\circ},\;89{}^{\circ},\;\ldots ,1{}^{\circ}\right\}$ were created and then corresponding RMSE and precision values were calculated for all 7 possible combinations of steering vector sets which are gathered in Table 3, at SNR = 10 dB and the obtained results are presented in Figure 11 and Figure 12.

As it can easily be observed in Figure 11, when a single set of steering vectors is used precision values deteriorate for low θ values and the best results can be produced for $\left\{V^{n_{mid}}\right\}$. For steering vector combinations involving at least 12 steering vectors, shown in Figure 11 and Figure 12, one can acquire precision lower than 19° for all possible θ angles, i.e., 0°<θ≤90°. Surprisingly, the most accurate results with precision lower than 11° for all θ angles can be obtained for the steering vector set $\left\{V^{n_{mid}},V^{n_{down}}\right\}$ containing 12 vectors. When the complete set $\left\{V^{n_{up}},V^{n_{mid}},V^{n_{down}}\right\}$ containing all 18 available steering vectors is used, the performance of generalized PPCC-MCP algorithm drops for θ∈(9°,33°).

**Figure 13:**
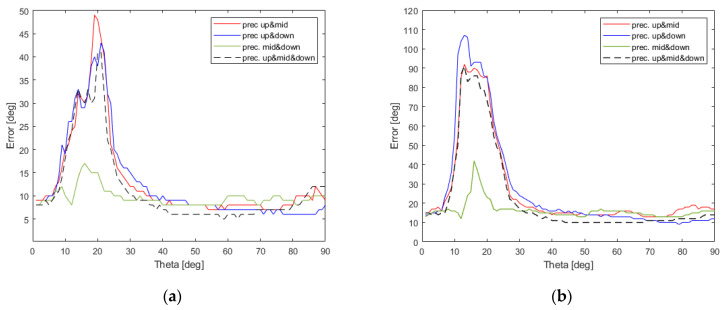
Comparison of precision values obtained using the proposed detailed DoA testing method and the combinations of steering vector sets giving the most accurate results for different SNR levels: (**a**) SNR = 5 dB (**b**) SNR = 0 dB.

To verify the performance of generalized PPCC-MCP algorithm for different SNR levels, two additional DoA tests, with SNR = 5 dB and SNR = 0 dB, were performed. As it can easily be noticed in Figure 13, the most accurate results in both cases can be obtained for only 12 steering vectors when $\left\{V^{n_{mid}},V^{n_{down}}\right\}$ steering vectors set is used. Therefore, two-row ESPAR antenna together with the proposed generalized PPCC-MCP algorithm can successfully be used for RSS-based DoA estimation in WSN nodes and gateways, even in noisy environments.

## 5. Conclusions

In this paper, we show how a two-row ESPAR antenna can be used for RSS-based DoA estimation. To this end, we have presented an analysis of all 18 available 3D antenna radiation patterns, being measured in an anechoic chamber, with respect to radiation coverage in the horizontal and vertical direction as well as their directional performance important in the estimation process. Moreover, we have proposed PPCC-MCP algorithm generalization that involves the usage of a very high number of calibration planes and can handle specific combinations of two-row ESPAR antenna radiation pattern sets. Detailed DoA estimation accuracy assessment, which involves thorough testing conducted along the elevation direction when RF signals impinging on the antenna arrive from arbitrary θ angles, shows that accurate results can be produced for only 12 steering vectors used. Precision lower than 11°, 17°, and 42° have been obtained for all θ angles when SNR was equal to 10 dB, 5 dB, and 0 dB, respectively. These results indicate that a two-row ESPAR antenna can produce accurate DoA estimation in the horizontal plane without prior knowledge about the elevation direction of the unknown RF signals even for low SNR values. Therefore, as the antenna beam switching in elevation and azimuth can be realized using a simple microcontroller and the DoA estimation relies solely on RSS values measured at the antenna output port, the antenna can easily be integrated with a WSN node or a WSN gateway. Moreover, 2D beamforming capabilities and accurate DoA estimation opens up new frontiers for ESPAR antenna applications involving new portable low-cost ground penetrating radars [44] and noninvasive microwave imaging devices supported by machine learning (ML) algorithms [45].

## Figures and Tables

**Table 1:** Measured parameters of the realized two-row ESPAR antenna.

Configuration	$\theta_{max}$	$G_{max}$	$HPBW_{\theta }$	$HPBW_{\varphi }$	$SLL_{\theta }$	$SLL_{\varphi }$	|S11|
UP	46°	7.7 dB	45°	88°	10 dB	14 dB	−20.9 dB
MID	52°	7.8 dB	43°	100°	10 dB	28 dB	−10.5 dB
DOWN	56°	8.1 dB	46°	93°	12 dB	25 dB	−8.8 dB

**Table 2:** Two-row ESPAR antenna steering vectors used in RSS-based DoA estimation.

n	$V^{n}$	Steering Vector Short Name	$\varphi_{max}$	$\theta_{max}$
1	[001101 010100]	$V_{UP}^{30}$	30°	46°
2	[100110 001010]	$V_{UP}^{90}$	90°	46°
3	[010011 000101]	$V_{UP}^{150}$	150°	46°
4	[101001 100010]	$V_{UP}^{210}$	210°	46°
5	[110100 010001]	$V_{UP}^{270}$	270°	46°
6	[011010 101000]	$V_{UP}^{330}$	330°	46°
7	[001010 100100]	$V_{DOWN}^{30}$	30°	56°
8	[000101 010010]	$V_{DOWN}^{90}$	90°	56°
9	[100010 001001]	$V_{DOWN}^{150}$	150°	56°
10	[010001 100100]	$V_{DOWN}^{210}$	210°	56°
11	[101000 010010]	$V_{DOWN}^{270}$	270°	56°
12	[010100 001001]	$V_{DOWN}^{330}$	330°	56°
13	[000100 001000]	$V_{MID}^{30}$	30°	52°
14	[000010 000100]	$V_{MID}^{90}$	90°	52°
15	[000001 000010]	$V_{MID}^{150}$	150°	52°
16	[100000 000001]	$V_{MID}^{210}$	210°	52°
17	[010000 100000]	$V_{MID}^{270}$	270°	52°
18	[001000 010000]	$V_{MID}^{330}$	330°	52°

**Table 3:** Combinations of steering vector sets used in verification of DoA estimation performance using generalized PPCC-MCP algorithm (see text for explanations).

Combination of Steering Vector Sets	Steering Vectors Numbers	Total Number of Steering Vectors
$\left\{V^{n_{up}}\right\}$	{1, 2, 3, 4, 5,6}	6
$\left\{V^{n_{mid}}\right\}$	{13,14,15,16,17,18}	6
$\left\{V^{n_{down}}\right\}$	{7,8,9,10,11,12}	6
$\left\{V^{n_{up}},V^{n_{mid}}\right\}$	{1, 2, 3, 4, 5,6, 13,14,15,16,17,18}	12
$\left\{V^{n_{up}},V^{n_{down}}\right\}$	{1, 2, 3, 4, 5,6, 7,8,9,10,11,12}	12
$\left\{V^{n_{mid}},V^{n_{down}}\right\}$	{13,14,15,16,17,18, 7,8,9,10,11,12}	12
$\left\{V^{n_{up}},V^{n_{mid}},V^{n_{down}}\right\}$	{1, 2, 3, 4, 5,6, 13,14,15,16,17,18, 7,8,9,10,11,12}	18

## Data Availability

Not applicable.

## References

[1] Zanella A., Bui N., Castellani A., Vangelista L., Zorzi M. (2014). Internet of things for smart cities. IEEE Internet Things J..

[2] Sotres P., Santana J.R., Sanchez L., Lanza J., Munoz L. (2017). Practical lessons from the deployment and management of a smart city internet-of-things infrastructure: The SmartSantander Testbed Case. IEEE Access.

[3] Low K.S., Win W.N.N., Er M.J. Wireless Sensor Networks for industrial environments. Proceedings of the International Conference on Computational Intelligence for Modelling, Control and Automation and International Conference on Intelligent Agents, Web Technologies and Internet Commerce (CIMCA-IAWTIC’06).

[4] Joshi G.P., Nam S.Y., Kim S.W. (2013). Cognitive radio Wireless Sensor Networks: Applications, challenges and research trends. Sensors.

[5] Al-Karaki J.N., Gawanmeh A. (2017). The optimal deployment, coverage, and connectivity problems in Wireless Sensor Networks: Revisited. IEEE Access.

[6] Tran T., An M.K., Huynh D.T. Symmetric connectivity in WSNs equipped with multiple directional antennas. Proceedings of the 2017 International Conference on Computing, Networking and Communications (ICNC).

[7] Brás L., Carvalho N.B., Pinho P., Kulas L., Nyka K. (2012). A review of antennas for indoor positioning systems. Int. J. Antennas Propag..

[8] Curiac D.-I. (2016). Wireless Sensor Network security enhancement using directional antennas: State of the art and research challenges. Sensors.

[9] Catarinucci L., Guglielmi S., Colella R., Tarricone L. (2014). Compact switched-beam antennas enabling novel power-efficient Wireless Sensor Networks. IEEE Sens. J..

[10] Groth M., Rzymowski M., Nyka K., Kulas L. (2020). ESPAR antenna-based WSN node with DoA estimation capability. IEEE Access.

[11] Lysko A.A. Towards an ultra-low-power electronically controllable array antenna for WSN. Proceedings of the 2012 IEEE-APS Topical Conference on Antennas and Propagation in Wireless Communications (APWC).

[12] Loh T., Liu K., Qin F., Liu H. (2014). Assessment of the adaptive routing performance of a Wireless Sensor Network using smart antennas. IET Wirel. Sens. Syst..

[13] Viani F., Lizzi L., Donelli M., Pregnolato D., Oliveri G., Massa A. (2010). Exploitation of parasitic smart antennas in Wireless Sensor Networks. J. Electromagn. Waves Appl..

[14] Skiani E.D., Mitilineos S.A., Thomopoulos S.C.A. (2012). A study of the performance of Wireless Sensor Networks operating with smart antennas. IEEE Antennas Propag. Mag..

[15] Ademaj F., Rzymowski M., Bernhard H.-P., Nyka K., Kulas L. (2021). Relay-aided Wireless Sensor Network discovery algorithm for dense industrial IoT utilizing ESPAR antennas. IEEE Internet Things J..

[16] Groth M., Nyka K., Kulas L. (2021). Calibration-free single-anchor indoor localization using an ESPAR antenna. Sensors.

[17] Harrington R. (1978). Reactively controlled directive arrays. IEEE Trans. Antennas Propag..

[18] Taillefer E., Hirata A., Ohira T. (2005). Direction-of-arrival estimation using radiation power pattern with an ESPAR antenna. IEEE Trans. Antennas Propag..

[19] Kulas L. (2018). RSS-based DoA estimation using ESPAR antennas and interpolated radiation patterns. IEEE Antennas Wirel. Propag. Lett..

[20] Plotka M., Tarkowski M., Nyka K., Kulas L. A novel calibration method for RSS-based DoA estimation using ESPAR antennas. Proceedings of the 22nd International Microwave and Radar Conference (MIKON).

[21] Asif M., Khan S., Ahmad R., Sohail M., Singh D. (2017). Quality of service of routing protocols in Wireless Sensor Networks: A review. IEEE Access.

[22] Shen J., Wang A., Wang C., Hung P.C.K., Lai C.-F. (2017). An efficient centroid-based routing protocol for energy management in WSN-assisted IoT. IEEE Access.

[23] Rzymowski M., Kulas L. RSS-based direction-of-arrival estimation with increased accuracy for arbitrary elevation angles using ESPAR antennas. Proceedings of the 12th European Conference on Antennas and Propagation (EuCAP 2018).

[24] Groth M., Kulas L. Accurate PPCC-based DoA estimation using multiple calibration planes for WSN nodes equipped with ESPAR antennas. Proceedings of the 2018 48th European Microwave Conference (EuMC).

[25] Cremer M., Dettmar U., Hudasch C., Kronberger R., Lerche R., Pervez A. (2015). Localization of passive UHF RFID tags using the AoAct transmitter beamforming technique. IEEE Sens. J..

[26] Huang S., Gan O.P., Jose S., Li M. Localization for industrial warehouse storage rack using passive UHF RFID system. Proceedings of the 2017 22nd IEEE International Conference on Emerging Technologies and Factory Automation (ETFA).

[27] Kulas L. (2017). Simple 2-D direction-of-arrival estimation using an ESPAR antenna. IEEE Antennas Wirel. Propag. Lett..

[28] Burtowy M., Rzymowski M., Kulas L. (2019). Low-profile ESPAR antenna for RSS-based DoA estimation in IoT applications. IEEE Access.

[29] Buffi A., Nepa P., Cioni R. SARFID on drone: Drone-based UHF-RFID tag localization. Proceedings of the 2017 IEEE International Conference on RFID Technology & Application (RFID-TA).

[30] De Oliveira M.T., Miranda R.K., Da Costa J.P.C.L., De Almeida A.L.F., De Sousa R.T. (2019). Low cost antenna array based drone tracking device for outdoor environments. Wirel. Commun. Mob. Comput..

[31] Rzymowski M., Kulas L. (2021). Two-row ESPAR antenna with simple elevation and Azimuth Beam Switching. IEEE Antennas Wirel. Propag. Lett..

[32] Duraj D., Rzymowski M., Nyka K., Kulas L. ESPAR antenna for V2X applications in 802.11p frequency band. Proceedings of the 2019 13th European Conference on Antennas and Propagation (EuCAP).

[33] Duraj D., Tarkowski M., Rzymowski M., Kulas L., Nyka K. RSS-based DoA estimation in 802.11p frequency band using ESPAR antenna and PPCC-MCP method. Proceedings of the 2020 23rd International Microwave and Radar Conference (MIKON).

[34] Lin Z., Lin M., de Cola T., Wang J.-B., Zhu W.-P., Cheng J. (2021). Supporting IoT with rate-splitting multiple access in satellite and aerial-integrated networks. IEEE Internet Things J..

[35] Lin Z., Lin M., Zhu W.-P., Wang J.-B., Cheng J. (2020). Robust secure beamforming for wireless powered cognitive satellite-terrestrial networks. IEEE Trans. Cogn. Commun. Netw..

[36] Lin Z., Lin M., Champagne B., Zhu W.-P., Al-Dhahir N. (2020). Secure beamforming for cognitive satellite terrestrial networks with unknown eavesdroppers. IEEE Syst. J..

[37] Lin Z., Lin M., Wang J.-B., De Cola T., Wang J. (2019). Joint beamforming and power allocation for satellite-terrestrial integrated networks with non-orthogonal multiple access. IEEE J. Sel. Top. Signal Process..

[38] Rahmat-Samii Y., Manohar V., Kovitz J.M. (2017). For Satellites, Think Small, Dream Big: A review of recent antenna developments for CubeSats. IEEE Antennas Propag. Mag..

[39] Sugiura S., Iizuka H. (2007). Reactively steered ring antenna array for automotive application. IEEE Trans. Antennas Propag..

[40] Zhang L., Gao S., Luo Q., Young P.R., Li Q. (2016). Planar ultrathin small beam-switching antenna. IEEE Trans. Antennas Propag..

[41] Xu B., Ying Z., Scialacqua L., Scannavini A., Foged L.J., Bolin T., Zhao K., He S., Gustafsson M. (2018). Radiation performance analysis of 28 GHz antennas integrated in 5G mobile terminal housing. IEEE Access.

[42] Hazmi A., Tian R., Rintamaki S., Milosavljevic Z., Ilvonen J., Van Wonterghem J., Khripkov A., Kamyshev T. Spherical coverage characterization of millimeter wave antenna arrays in 5G mobile terminals. Proceedings of the 2019 13th European Conference on Antennas and Propagation (EuCAP).

[43] Groth M., Leszkowska L., Kulas L. Efficient RSS-based DoA estimation for ESPAR antennas using multiplane SDR calibration approach. Proceedings of the 2018 IEEE-APS Topical Conference on Antennas and Propagation in Wireless Communications (APWC).

[44] Das U., Boer H.J., Van Ardenne A. Phased array technology for GPR antenna design for near subsurface exploration. Proceedings of the 2nd International Workshop onAdvanced Ground Penetrating Radar.

[45] Salucci M., Gelmini A., Vrba J., Merunka I., Oliveri G., Rocca P. (2018). Instantaneous brain stroke classification and localization from real scattering data. Microw. Opt. Technol. Lett..

